# “Keeping the Boogie Man Away”: Medication Self-Management among Women Receiving Anastrozole Therapy

**DOI:** 10.1155/2012/462121

**Published:** 2012-12-27

**Authors:** Karen Wickersham, Mary Beth Happ, Catherine M. Bender

**Affiliations:** ^1^School of Nursing, University of Maryland, Baltimore, 655 West Lombard Street, Room 731A, Baltimore, MD 21201, USA; ^2^College of Nursing, The Ohio State University, 378 Newton Hall, 1585 Neil Avenue, Columbus, OH 43210, USA; ^3^Department of Health and Community Systems, School of Nursing and University of Pittsburgh Cancer Institute, University of Pittsburgh, 415 Victoria Building, Pittsburgh, PA 15261, USA

## Abstract

The oral hormonal agent anastrozole improves clinical outcomes for women with breast cancer, but women have difficulty taking it for the five-year course. The unique medication-taking experiences related to self-management of anastrozole therapy for women with early stage breast cancer are not known. Our purpose was to describe the medication-taking experiences for postmenopausal women with early stage breast cancer who were prescribed a course of anastrozole therapy. Twelve women aged 58 to 67 years, midway through therapy, participated in audio-recorded interviews. Women's medication-taking experiences involved a belief in their importance and an imperative to take anastrozole. We found that women's side effect experiences, particularly menopausal symptoms, were significant, but only one woman stopped anastrozole due to side effects. Medication-taking included routinization interconnected with remembering/forgetting and a storage strategy. Some women noted a mutual medication-taking experience with their spouse, but most felt taking anastrozole was something they had to do alone. Our results provide insight into the way some women with early stage breast cancer manage their hormonal therapy at approximately the midpoint of treatment. Next steps should include examinations of patient-provider communication, potential medication-taking differences between pre- and postmenopausal women, and the effects of medication-taking on clinical outcomes.

## 1. Introduction

Treatment of cancer has shifted to greater use of oral cancer agents [1], transferring responsibility for medication management to the patient. Therapy with oral aromatase inhibitors (AIs) like anastrozole has been shown to improve clinical outcomes for postmenopausal women with early stage breast cancer [2]; however, women have difficulty taking their medication for the generally prescribed five-year course. Oral AI therapy is a “chronic” care cancer treatment prescribed to prevent recurrence, but most women receiving this treatment do not have active cancer. 

Self-management has been defined as one's ability to manage symptoms, treatment, physical and psychosocial consequences, and lifestyle changes that are fundamental to living with a chronic condition such as cancer [3]. Self-management for postmenopausal women with early stage breast cancer includes medication-taking with oral AI therapy, which requires women to perform activities such as identifying and counting pills, timing of pill taking, and obtaining and refilling prescriptions [4]. Qualitative inquiry provides unique information concerning the medication-taking experiences for patients with chronic disorders [4–6], but little research has focused on the medication-taking experiences for patients with cancer. Ersek et al. [7, 8] explored the reasons patients with cancer have trouble taking their pain medication; however, the purpose of analgesia is different from that of medication for prevention of recurrence of breast cancer. The two published studies examining medication-taking for patients with cancer have been conducted for children or adolescents with leukemia [9, 10], who have different issues related to medication-taking including developmental concerns such as egocentrism, concrete thinking, and parental involvement [10]. Thus, to address this gap in the literature, our purpose was to describe the medication-taking experiences of postmenopausal women with early stage breast cancer who were receiving the oral hormonal agent, anastrozole. We sought to answer the question: “What are the experiences of women who take anastrozole therapy?”

## 2. Methods

We wished to learn *how *women with early stage breast cancer managed their anastrozole therapy daily [11], rather than whether or not they took it as prescribed [12]. As such, we used qualitative description to generate a complete narrative of the medication-taking experiences of women with early stage breast cancer who are prescribed anastrozole therapy [13]. Qualitative description as a method guides analysis and interpretation of data to produce findings that are close to the data, which is appropriate when little research about a particular phenomenon exists.

### 2.1. Parent Study

We accessed an existing sample and data from an ongoing study, The Anastrozole Use in Menopausal Women (AIM) Study, which examines the effect of anastrozole on cognitive function in women with early stage breast cancer (“The AIM Study”). The AIM Study includes postmenopausal women less than 75 years old who speak and read English and have completed at least eight years of education. Women are excluded for self-reported hospitalization for psychiatric illness within the last two years, prior diagnosis of other cancers and neurologic illness (e.g., stroke, multiple sclerosis, dementia syndrome), and distant metastases. Women are followed for their five-year course of hormonal therapy. Adherence to anastrozole is assessed continuously using an electronic Medication Event Monitoring System (MEMS) (AARDEX, Ltd.). MEMS is a bottle cap with a microprocessor that records the date and time of each cap opening from a standard medication bottle (i.e., not the weight of the bottle). Access to The AIM Study participants allowed us a unique opportunity to answer our research question about the experience of medication-taking and full scope of medication-taking behaviors in this sample.

### 2.2. Current Study Procedure

The University of Pittsburgh Institutional Review Board approved the “current study,” a followup to The AIM Study. At the time we began our study (2009), we had complete six-month data for 47 women. The AIM Study PI sent mailings to these 47 women to determine their interest in participating in the follow-up study. We interviewed all 12 women who responded to the mailings. Interested women contacted the researcher (KW), who then described the purpose of the interviews by telephone. All women provided written informed consent prior to their interview. 

### 2.3. Interviews

The researcher (KW) performed in-depth semistructured interviews (*n* = 12), averaging 30 to 40 minutes in length, using an interview guide of open-ended questions adapted from two previous qualitative studies of medication-taking [6, 14]. Questions included asking women about what it was like to take anastrozole, how and why they began taking it, how it made them feel, how it was different from their previous treatments, how they took it on a typical day and the strategies they used, what they found difficult about taking anastrozole, forgetting to take it, and who helped them manage their medication. Interviews conducted at a convenient private location (*n* = 6) were shorter in duration, but not less informative, than those performed in the participant's home (*n* = 6). Interviews were audio-recorded with observational notes for recording of the participant's nonverbal cues and eye contact [13]. Participants received $10 upon completion of the interview. 

### 2.4. Sociodemographic and Clinical Data

The following demographic and clinical data available from The AIM Study database were used to describe the sample. Sociodemographic information was collected using the University of Pittsburgh, School of Nursing, Center for Research in Chronic Disorders Sociodemographic Questionnaire. Women's depressive symptoms were measured using the Beck Depression Inventory-II (BDI-II) [15]. Anxiety was assessed with the Profile of Mood States (POMS) Tension-Anxiety subscale [16]. Information concerning stage of breast cancer, tumor type, radiation therapy, and chemotherapy was abstracted from the patient medical record. Side effects of hormonal therapy were assessed with the Breast Cancer Prevention Trial (BCPT) Symptom Checklist [17, 18]. BDI-II, POMS, and BCPT data from The AIM Study 6-month and 18-month time points (closest to the interviews with the most complete data) were used for the analysis.

As another form of description of medication-taking of anastrozole, we categorized women according to their MEMS cap adherence rate: 100% adherers, good-adherers (90–99%), adequate-adherers (80–89%), and low-adherers (below 80%). These categories were based on the literature [14, 19, 20]. We defined adherence as the percent of the prescribed doses taken. Women who discontinued or who were switched to another AI by their oncologist due to toxicities were included, because therapy discontinuation is an important variation (and perhaps consequence) of medication-taking.

### 2.5. Data Analyses

The researcher (KW) reviewed each transcript while listening to the audiotape with observational notes for accuracy and for an understanding of the participant's focus. All interviews were transcribed in a word document and then uploaded into ATLAS.ti (6.2.27) (ATLAS.ti Scientific Software Development GmbH, Berlin, Germany) to manage and organize the data. Observational notes were summarized and included with each transcript. We developed a timeline for each woman that outlined the timing of her breast cancer diagnosis, the start of anastrozole, and the side effects she experienced after beginning anastrozole to gain a sense of her overall experience with this treatment. As analysis progressed, interview language was refined for clarity. Probes were added about forgetting to take medication (e.g., “How did you realize you forgot?”), unexpected events that affected medication-taking (e.g., vacation/travel), and information received at therapy initiation (e.g., “What were you told about Arimidex®?”).

Descriptive statistics were computed using IBM SPSS Statistics v.20.0 (Armonk, NY) to describe the data distributions and to characterize the study sample. We used the independent samples *t*-test (or Mann-Whitney *U*-test, if data were nonnormal) and the Chi-square test of independence (or Fisher exact test, if cell sizes were sparse) to compare women in the current study with women in The AIM Study to investigate whether there were any significant differences between the two groups.

Qualitative content analysis [21] was the primary method for data analysis. For each interview, the researcher (KW) examined the data line by line to label (open code) text related to the women's medication-taking experiences, using the interview questions as a guide. Similar codes were grouped into categories, which were examined for central themes. Dimensional analysis was applied to themes to detect variations, specificity, and range [22]. Matrices were constructed for comparison and pattern recognition of participant characteristics (sociodemographic, breast cancer type and treatment, adherence level), side effects, depressive symptoms, and anxiety, merging qualitative data, and quantitative measures (BCPT, BDI-II, POMS). Numerical counts were used to characterize the strength of the main themes and subthemes within each case [23]. In this report, we use “most” to describe occurrence of a theme in at least nine women. Turning points in the analysis included the realization that medication-taking occurred despite side effect presence and severity and the pervasiveness of fear of breast cancer recurrence. Sampling, interviewing, and analysis continued until we reached informational redundancy, that is, no new themes or patterns were recognized (*n* = 9); at that time, we enrolled three women for further sample diversity and to confirm existing findings. No new themes emerged and we achieved informational redundancy, but we cannot claim full saturation due to limited access to low-adherers and women who discontinued therapy.

 We implemented the following steps to assure the trustworthiness of the data, analysis, and research process. (a) A Co-Investigator (MBH) with expertise in qualitative methods and medication-taking research [6] audited the data by performing dual coding and review of the data to ensure the credibility of the analysis. (b) Four members of a weekly group analysis meeting discussed data exemplars, coding, and analytic decisions. (c) Four follow-up telephone interviews were performed to further clarify developing themes. For example, when several women mentioned that they had friends or relatives who were prescribed anastrozole and were no longer taking it, a follow-up question was added to further explore this experience and key informants (i.e., provided rich data about the selected subthemes) were recontacted to clarify this theme. (d) All interview data, notes, and memos were documented using ATLAS.ti (6.2.27) (ATLAS.ti Scientific Software Development GmbH, Berlin, Germany) software.

## 3. Results

### 3.1. Participant Characteristics

Twelve women aged 58 to 67 years were interviewed between June 2009 and April 2010. All women were white and well educated, and were similar to the women who participated in The AIM Study (98.1% white) (Table 1). Eleven women had been taking anastrozole for two and one-half to three years at the time of their interview. One woman discontinued anastrozole after six months due to arthralgias (hip pain) and was then switched to another AI by her oncologist. At the time of her interview, she had discontinued all AI therapy due to side effects. Women in the current study had six-month adherence levels ranging from 38.4% to 100% (mean = 87.8%), which were similar to women participating in The AIM Study (mean = 88.1%).

In their interviews, the women shared their perceptions about anastrozole (“what I think”), their experiences with side effects and side effect severity (“how it makes me feel”), and their day-to-day self-management of anastrozole (“what I do”). These three main topical categories describe the women's engagement in self-management of anastrozole and represent key dimensions of self-management in this early phase of breast cancer survivorship. These categories involved an overarching belief in the importance of anastrozole, as well as an imperative to take it. We found that though the women's side effect experiences were significant, only one woman stopped taking anastrozole due to side effects. The women's descriptions of their day-to-day medication-taking experiences with anastrozole included barriers and facilitators to taking anastrozole daily. 

### 3.2. Perceptions about Anastrozole—“What I Think”: Keeping the Boogie Man Away

All women assigned a sense of the value, purpose, or importance to anastrozole that offset other challenges associated with managing anastrozole, including side effect severity. The importance of anastrozole was defined as a woman's awareness or beliefs about therapy, the value, benefit, or relative worth of taking anastrozole, and her commitment or motivation to take anastrozole. Most women remained motivated to take anastrozole despite the side effects they experienced:

*I still take it. I still take it... if I thought that the medication was going to make me have early- onset dementia, I would think about it more, and I do know there've been some thoughts about that, but I still take it. I don't want to, (lowers tone) get breast cancer again, so, I take it.*

When discussing their beliefs and motivation to take anastrozole, most women used imagery rather than the term “cancer recurrence”: “I'm taking it to keep the boogie man away.” Another took it to keep “loose cells [from] travelling where they shouldn't.” Another woman said, “That's very important, that pill... I want to live... I want to stay healthy.” One participant with 100% adherence described a heightened consciousness about the role of anastrozole:

*I was conscious of saying, “Okay, do your job in there, Arimidex®.”... it was a funny thing. I didn't experience that in the first year and maybe only because I was experiencing those other things [side effects]. But, there was this short period of time where I'd take my water, drink it down and say, “Okay, do your thing, Arimidex®, get in there, kill any cells that you see...”*

Conversely to the above description, two women indicated that it was “no big deal” to take or to miss a dose of anastrozole, presumably meaning that missing one dose would not harm her overall outcome.

*So I think if you're taking Arimidex® over years, they're [adrenal glands] not going to all of a sudden, if you miss one, they're not going to all of a sudden get back going again when they've been put to sleep as... for as long as they have been... I mean if you skipped a whole month... or even a whole week... that might be a different story...' cause then they'd start getting their act back together.*

Although all women were motivated to take anastrozole and recognized its value, some interviews suggested tension between the desire to prevent cancer recurrence and uncertainty about taking anastrozole. This woman's comments further revealed ambiguity regarding the value of the medication in preventing cancer recurrence:

*To me, the benefit of not getting cancer, whether it's breast or some other site, is certainly more advantageous than putting up with a little bit of wrinkles or some other problem... but on the other hand, you wonder.*

Women further indicated that there was a necessity or obligation to take anastrozole that went beyond their belief in its importance. This treatment imperative included her commitment to the program and “wanting to get to the finish line.” The imperative was self-motivated, “I would never dream of quitting” “I truthfully want to do the five years. I want to complete the program as is,” or externally motivated from a relative, a friend, or a health care provider, “My mom... would push me to take it and say ‘you need to continue on this.” 

Several women mentioned an imperative to take anastrozole based “on doctor's orders.” “He told me that I'd have to take it, and so I took it.” Women were told by their health care provider (HCP) to take anastrozole daily, but they were they given no other instructions. The women expressed willingness to discuss their side effects with their oncologist or HCP; however, they were rarely asked about their experiences. In some cases, they received conflicting advice from HCPs. For example, when discussing her foot pain, one woman indicated, “Foot doctor says no [unrelated to anastrozole].” Everyone else says “Ah, yeah.” Furthermore, when some women desired specific information about anastrozole, such as what time of day to take the medication, they asked their pharmacist (or chemist).

### 3.3. Side Effects and Side Effect Severity—“How It Makes Me Feel”: Being Thrown Back into Menopause

For all women, the opening question (“Tell me about your experience taking anastrozole”) led without prompting to a description of the side effects of anastrozole. All women immediately described challenges with hot flashes and associated sleep disturbances, arthralgias, fatigue, “female things”, weight gain or loss, and struggles with forgetfulness or memory loss, regardless of their MEMS cap adherence level. The women described the timing of when side effects occurred in relation to starting anastrozole (e.g., within a few months or right away), the time of day the side effects occurred, and the duration of the side effects (e.g., lasting a few minutes). They described how the side effects affected their daily life or altered their lifestyle, characterization (e.g., “like a torch”), frequency (e.g., occurring every few hours), and their attribution that the side effect was due to anastrozole, another therapy, or a process such as aging. One woman experiencing menopausal-like symptoms stated that anastrozole “threw me back into menopause.” Another woman characterized her hot flashes:

*Overall it feels like a torch... the chest area and face and forehead; my forehead's like soaking wet now... they come on real fast and last about a minute or two... during the night I might wake up it seems every two hours... like at midnight, two o'clock, four o'clock, six o'clock, and you know it wakes me up and sometimes I can't go back to sleep so that is an additional problem.*

One woman who took anastrozole in combination with chemotherapy described how she felt about her experiences with memory problems:

*The only thing I do have a problem with, and I have noticed it, is my memory. Now I'm remembering a lot of things... today, talking to you, but if somebody said, “Well, I told you that yesterday,” or “Don't you remember I...” “I can't remember.” I have to really think, and that scares me. I mean I had a bad memory before (laughs)... but it, it is worse. It is, it is worse.*

The woman who discontinued AI therapy due to hip pain described the related uncertainty of the underlying cause of her pain:

*I think once you have cancer you start to think, “Is this mets to the bone, or is this mets somewhere else... or is it a side effect from the medication”…when I take medication, I try not to read the side effects unless I'm having problems and then I go to the side effects and say, “Ah, yeah, maybe this is it.” But when I started... in my hips, and it was at night and I was having trouble sleeping, I just decided that... this [anastrozole] wasn't for me.*

To further explore the problem of the women's side effects and side effect severity, we constructed profiles of side effects for each participant by combining those side effects reported in interviews with information from the BDI-II, POMS, and BCPT (Table 2). Participants reported three to six side effects; most women (*n* = 10) reported five or more side effects. The two women classified as “low-adherers” reported the same type and number of side effects as the two 100% adherers. The woman who discontinued AI therapy due to side effect severity was classified as a “good” adherer and reported the same five side effects as the other women. Women expressed varying levels of depressive symptoms and anxiety when completing the BDI-II and POMS surveys; however, only one woman expressed these symptoms during her interview, and her scores did not indicate depression or anxiety. This mixed data analysis revealed no patterns between symptom number, type, and severity and adherence category.

Generally, women used pharmaceuticals (e.g., antidepressants), physical therapy, or other daily management or compensation strategies to alleviate the side effects they experienced. For example, one woman avoided or limited her activities, while another wrote down tasks or names to remember them. The woman with “summertime blues” described her frustration with word-finding problems and how she compensated:

*I've worked with lots of women, and we all say (laughter) estrogen, the menopausal breakdown. But, I have days when I just... can't remember things like names or specific words for thoughts... And I'm usually really good. I love words, and I'm usually pretty good with them. But, I just have days when I can't, and I'm not as articulate... I just finished helping with the summer camp and we had about 18 college counselors... I remembered all their names, and once in a while I'd completely blank... but I had a notebook, I had my cheat sheet.*

### 3.4. Day-to-Day Self-Management—“What I Do”: Doing It Yourself

#### 3.4.1. Barriers

All women described in detail the actual hand-to-hand, tangible characteristics of taking anastrozole. Many women mentioned that the pill was tiny and easy to swallow. However, when anastrozole was packaged in blister packs for a short time (e.g., a few months), the women expressed extreme difficulty and irritation with opening the blister pack. One woman who received two three-month supplies of anastrozole packaged in blister packs described how the packaging affected her daily medication-taking of anastrozole:

*... My husband had to get them out... Arimidex® people ought to know that that is not acceptable. (Laughter) Maybe they found that out... but I'll tell you that was the only time that I considered stopping. Because I have arthritis in my hands... and they're old hands... it was very, very difficult. I couldn't put it through, you know, so I tried to use a penknife, I tried to flip up the little foil thing... and sometimes I'd try to slice off the bubbles like this. (Gestures). It's just hard. I couldn't do it.*

#### 3.4.2. Facilitators

 Central to self-management of anastrozole for all of the women was the routinization or integration of anastrozole into their everyday lives as anastrozole-taking became a consistent, accepted, or habitual medication self-management practice. They described timing anastrozole administration with meals or other medications, associating it with a visual cue (e.g., seeing the bottle on the window sill), a central location (e.g., kitchen), or a storage strategy (e.g., weekly pill minder). Women stated that participation in The AIM Study helped to routinize their medication-taking practice. Most (*n* = 11) were already taking other prescription medications, vitamins, or supplements and incorporated anastrozole within their established routine.

Associated with routinization was remembering/forgetting to take anastrozole and their realization, reaction, and strategies for taking anastrozole after forgetting a dose:

*I was in that AIM Study and I had the little bottle, and I swear I took it every day, but there was a few times when she (study nurse) put it on the little machine to see that I had missed it a few times. Now last night I went to bed and I remembered about 1:00 [AM] and I came down the steps and took it.*

Frequently, remembering was linked to a certain time of day or a storage strategy, such as a weekly pill container. For example, one woman stated she did not forget to take her anastrozole “Cause I take it with my morning vitamin, my calcium, and fish oil.” Another woman described:

*When I started it, that's when I put into my day [pill minder]... I've had no trouble remembering to take it, and that seems to be a good time [after supper] since its after my work day, except when I have a meeting, I don't forget.*

Although women felt they remembered to take anastrozole, over half stated they occasionally missed a dose, only realizing it when noticing the pill was still in the container or her pocket, or when the MEMS cap was downloaded. Both low adhering women discussed the management of their missed doses in a similar manner.

*Sometimes I might play the 12 hour shuffle if I know I didn't take it the night before... maybe I'll take it in the morning, and then at bedtime, so it's probably putting two in one day, but trying to spread them apart, so it's not quite the same.*

Some women described medication-taking as a social or a “mutual medication-taking” experience, referring to taking anastrozole at the same time a spouse or other family member took their own medications. Several women mentioned that they had friends or relatives who were prescribed anastrozole and were no longer taking it, but they denied that this deterred them from taking their own anastrozole. Most women described a “solitary” experience in which no one can or needs to help with taking anastrozole. A woman who lived alone stated, “I just have to do it.” Another stated, “... I don't think he [husband] thinks about me taking my medication at all.” 

## 4. Discussion

Our purpose was to describe the medication-taking experiences of postmenopausal women with early stage breast cancer who were receiving anastrozole therapy. The women's engagement in the self-management of anastrozole involved a predominant belief in the importance of anastrozole, as well as an imperative to take it. We found that though their side effect experiences were significant, the women remained motivated to take anastrozole; only one woman stopped taking anastrozole due to side effects. All medication-taking practices were facilitated by routinization that was interconnected with remember/forgetting to take anastrozole and a storage strategy (e.g., pill minder). Some women noted a mutual medication-taking experience with their spouse, but most felt that taking anastrozole was something they had to manage alone.

Little research has addressed motivation to take oral AI therapy for women with breast cancer. For example, The ATAC Trialists' Group [2] found that fewer women withdrew from therapy with anastrozole when compared to tamoxifen, but the reasons for discontinuation were not reported. In a qualitative comparison of 13 stroke patients who were classified as high- and low-adherers, Chambers and colleagues [24] found that both groups reported intentional and nonintentional adherence. Although some low-adherers in Chambers' [24] study reported occasionally skipping a medication, stability of a medication routine and beliefs about medication were central themes describing medication self-management in our sample. Pound and colleagues [25] discussed in a metasynthesis of qualitative studies of lay medication-taking experiences that little research focuses on men and women who reject their medications or take their medications without questioning. Our results suggest that some women who take anastrozole without question may do so because they believe in the medication's value and importance. Furthermore, the use of imagery and personification in many of the women's speech is additional evidence of the value and power that the women assigned to anastrozole. 

The side effects the women reported were consistent with reports of menopausal symptoms induced by breast cancer treatment [26, 27], as well as with previous qualitative research describing women's experiences with hot flashes, the impact of hot flashes on daily life, and the higher priority that women placed on breast cancer treatment over menopausal symptoms [28–30]. Garreau and colleagues [31] found that women receiving AIs switched therapy more often (47.5%) than those taking tamoxifen (37%). Sedjo and Devine [32] found that 30% of women discontinued AI therapy; of those, 84% discontinued due to side effects.

Given these findings, we would have expected women in the current study to describe switching or discontinuing AI therapy more often, but 11 of 12 women indicated that side effects did not deter them from taking anastrozole. The fact that the women who were lower-adherers reported the same type and number of side effects as women who were 100% adherers is interesting and suggests that side effects related to AI therapy are significant to women midway through treatment. The impact of side effects on the medication-taking process with AI therapy for breast cancer prevention requires further examination. It is possible that completion of the BCPT, POMS, and BDI II surveys may have primed the women to describe the side effects that they felt were the most important, most persistent, and/or most present. 

 The manner in which women logistically and socially managed anastrozole therapy was consistent with research examining medication-taking for patients with chronic conditions [4, 6, 32, 33]; however, in our study, mutual-medication taking went beyond social support or reminding or assisting patients with their medications [4]. Rather, it included a partnership with a spouse in the physical taking of anastrozole that was part of her daily routine. Furthermore, self-management of medications often involves coordination between the patient and the health care team, but in the current study, the women received little in the way of instructions concerning medication use, side effects, and daily management of anastrozole. This suggests that patient-provider communication and information provision are potentially unmet important needs for women taking anastrozole therapy.

The most significant limitation in the current study is the potential influence of participation in The AIM Study. The women who participated in the interviews were neither naïve to research nor to anastrozole adherence, which may have affected their responses. We interviewed all women who responded to the mailings, but most of the women were already successful in self-management of anastrozole. Women may have had difficulty recalling their early experiences taking anastrozole, or they may have been primed to discuss side effects due to the recent completion of The AIM Study surveys. 

All women in the current study were white and well educated. Although the women we interviewed were representative of the women who took anastrozole in The AIM Study, they may not represent postmenopausal women with early stage breast cancer in the general population. Racial/ethnic disparities in treatment may affect self-management of medication and should be investigated.

Qualitative description allows one to fully describe a particular phenomenon; however, our sampling strategy did not allow us to obtain a complete picture of the women's process of taking anastrozole. In the current study, the women were approximately midway through their five-year course of therapy; therefore, they may have been more established in their medication-taking routines and less likely to discontinue anastrozole therapy. Interviewing women at earlier points in their treatment may help elicit the full scope of how side effects of hormonal therapies affect medication-taking. 

The women in the current study were mostly 100%- or good-adherers. While we reached information redundancy in our sample, we did not saturate with regard to those who were low-adherers or who had stopped AI therapy. We were able to interview one participant who had discontinued anastrozole due to hip pain; however, we may have missed women at the beginning of their treatment who discontinued or were switched to other AIs. Nonetheless, our results provide a snapshot of the way some women at approximately the midpoint of their hormonal therapy manage their medications, and thus may inform interventions that would aid them in completing the full five years of anastrozole therapy.

## 5. Conclusion

The women's experiences suggest several implications for clinical practice concerning medication self-management. Given that women were offered minimal information about taking anastrozole therapy, provision of information about anastrozole, its side effects, and how and when to take it may be beneficial, beginning with the first clinic visit with ongoing reassessment at subsequent clinic visits. Second, while most women indicated they experienced similar side effects, the trajectory of those side effects differed among the women. This suggests that ongoing side effect assessment by the health care team is needed even after therapy is well established. Finally, questions focusing on the patient's medication-taking experiences as a whole, rather than an overall verbal assessment of adherence, may prompt further discussion, including why they do or do not take their medication.

Our study offers a unique perspective into the medication-taking experiences of some postmenopausal women with early stage breast cancer who were midway through a course of anastrozole who were successful at self-management of anastrozole therapy. While reports examining the end result of self-management of medication (adherence) have been published, reports of research explaining how women view their experiences taking oral hormonal therapy are lacking. Our results help explain why some women, regardless of their measured adherence level, take anastrozole therapy without question and continue despite the side effects of anastrozole. Next steps should include investigations of medication-taking about: (a) women who are low-adherers to AI therapy; (b) socioeconomically and ethnically diverse patient samples; (c) potential differences between pre- and postmenopausal women, particularly side effect severity and medication-taking; (d) effects of medication-taking on clinical outcomes; and, (e) women with breast cancer taking oral targeted therapies. 

## Figures and Tables

**Table 1:** Participant sociodemographic, breast cancer, and breast cancer treatment characteristics.

Characteristic	Current study participants (*n* = 12)	The AIM Study women who received anastrozole (*n* = 162)	*P* value
Age (in years) mean	62.5	60.1	.729
Years of education mean	14.8	15.1	.844
Marital status n (%)			.528
Married	6 (50.0)	109 (67.3)	
Divorced	2 (16.7)	20 (12.3)	
Never married	3 (25.0)	18 (11.1)	
Widowed	1 (8.3)	13 (8.0)	
MEMS 6-month adherence %	87.8	88.7	.129
Breast cancer treatment n (%)			
Radiation therapy	11 (91.7)	32 (19.8)	.874
Mammosite therapy	1 (8.3)	12 (7.4)	
Chemotherapy with anastrozole	2 (16.7)	25 (15.4)	.025*

*P < .05.

**Table 2:** The women's self-reported side effects.

ID	Hot flashes	Arthralgias	Sleep disturbance	Fatigue	Weight gain or loss	Anxiety/depressive symptoms	“Female things”	Cognitive problems
1	B	Both		I	B	BDI-II, P		B
2		Both	I		B	BDI-II, P	Both	Both
3*		Both			B	BDI-II, P, I	BCPT	I
4	Both		I		B	BDI-II, P	Both	B
5	B				Both	BDI-II, P		B
6	Both	B	I			BDI-II, P	B	B
7	Both	B			B	BDI-II, P	B	B
8	Both	B	I	I	Both	BDI-II, P		Both
9	Both	B	I	I	B	BDI-II, P		B
10	Both	Both	I			BDI-II, P	B	B
11	B	Both			B	BDI-II, P	B	B
12	Both	Both	I	I		BDI-II, P		Both

Arthralgias were defined as aches, pains, and joint pains. “Female things” were defined as vaginal itching, vaginal bleeding, vaginal discharge, or pain with intercourse.

B: Symptoms reported by the participant on the Breast Cancer Prevention Trial (BCPT) Symptom Checklist only.

I: Symptoms reported by the participant during the interview only.

Both: Symptoms reported by the participant in both the interview and the BCPT.

P: Anxiety reported by the participant in the Profile of Mood States (POMS) Tension-Anxiety Subscale.

BDI-II: Depressive symptoms reported by the participant in the Beck Depression Inventory-II Scale.

*This participant was the only woman to specifically express depressive symptoms or anxiety in her interview.
